# Australasian ACPGBI risk prediction model for 30‐day mortality after colorectal cancer surgery

**DOI:** 10.1002/bjs5.50356

**Published:** 2020-09-28

**Authors:** S. Wilkins, K. Oliva, E. Chowdhury, B. Ruggiero, A. Bennett, E. J. Andrews, O. Dent, P. Chapuis, C. Platell, C. M. Reid, P. J. McMurrick

**Affiliations:** ^1^ Cabrini Monash University Department of Surgery Malvern Victoria; ^2^ Department of Anaesthesia Cabrini Hospital Malvern Victoria; ^3^ Department of Epidemiology and Preventive Medicine, School of Public Health and Preventive Medicine Monash University Melbourne Victoria; ^4^ School of Public Health Curtin University Perth Western Australia; ^5^ Colorectal Surgical Unit St John of God Subiaco Hospital, University of Western Australia Perth Western Australia; ^6^ Department of Colorectal Surgery Concord Hospital Sydney New South Wales Australia; ^7^ Discipline of Surgery, Sydney Medical School University of Sydney Sydney New South Wales Australia; ^8^ Bi‐National Colorectal Cancer Audit Cork Ireland; ^9^ Department of Surgery Cork University Hospital Cork Ireland

## Abstract

**Background:**

Postoperative mortality after colorectal cancer surgery varies across hospitals and countries. The aim of this study was to test the Association of Coloproctologists of Great Britain and Ireland (ACPGBI) models as predictors of 30‐day mortality in an Australian cohort.

**Methods:**

Data from patients who underwent surgery in six hospitals between 1996 and 2015 (CRC data set) were reviewed to test ACPGBI models, and patients from 79 hospitals in the Bi‐National Colorectal Cancer Audit between 2007 and 2016 (BCCA data set) were analysed to validate model performance. Recalibrated models based on ACPGBI risk models were developed, tested and validated on a data set of Australasian patients.

**Results:**

Of 18 752 patients observed during the study, 6727 (CRC data set) and 3814 (BCCA data set) were analysed. The 30‐day mortality rate was 1·1 and 3·5 per cent in the CRC and BCCA data sets respectively. Both the original and revised ACPGBI models overestimated 30‐day mortality for the CRC data set (observed to expected (O/E) ratio 0·17 and 0·21 respectively). Their ability to correctly predict mortality risk was poor (*P* < 0·001, Hosmer–Lemeshow test); however, the area under the curve for both models was 0·88 (95 per cent c.i. 0·85 to 0·92) showing good discriminatory power to classify 30‐day mortality. The recalibrated original model performed well for calibration and discrimination, whereas the recalibrated revised model performed well for discrimination but not for calibration. Risk prediction was good for both recalibrated models. On external validation using the BCCA data set, the recalibrated models underestimated mortality risk (O/E ratio 3·06 and 2·98 respectively), whereas both original and revised ACPGBI models overestimated the risk (O/E ratio 0·48 and 0·69). All models showed similar good discrimination.

**Conclusion:**

The original and revised ACPGBI models overpredicted risk of 30‐day mortality. The new Australasian calibrated ACPGBI model needs to be tested further in clinical practice.

## Graphical abstract

The aim was to test the Association of Coloproctologists of Great Britain and Ireland (ACPGBI) original (2003) and revised (2010) models as predictors of 30‐day mortality in an Australian patient cohort, and then recalibrate these models using data from Australian patients. This study included 10 541 patients treated over a 20‐year interval in Australia and New Zealand. The ACPGBI models overestimated 30‐day mortality, and so both ACPGBI models should be used with caution in Australia.

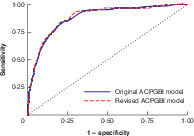

Mortality risk prediction after colorectal surgery

## Abstract (es)

**Antecedentes:**

La mortalidad postoperatoria tras la cirugía del cancer colorrectal (*colorectal cáncer*, CRC) varía entre hospitales y países. El objetivo de este estudio era evaluar los modelos de la Asociación de Coloproctólogos de Gran Bretaña e Irlanda (*Association of Coloproctologists of Great Britain and Ireland*, ACPGBI) como predictores de mortalidad a los 30 días en una cohorte de pacientes de Australia.

**Métodos:**

Se revisaron los datos de pacientes sometidos a cirugía en seis hospitales entre 1996‐2015 (datos CRC) para evaluar los modelos ACPGBI, mientras que los datos recogidos en 79 hospitales en la auditoría bi‐nacional de cáncer colorrectal (*Bi‐National Colorectal Cancer Audit*) entre 2007‐2016 (datos BCCA) se analizaron para validar el comportamiento del modelo. Se desarrollaron modelos recalibrados basados en los modelos de riesgo ACPGBI que fueron aplicados y validados en un conjunto de datos multi‐institucionales de pacientes australianos. La mortalidad observada y estimada (tasa 0/E) a 30 días se calculó en los modelos ACPGBI original y revisados usando el test de Hosmer‐Lemeshow y los análisis de la curva de las características operador‐receptor (ROC) para evaluar la calibración y discriminación de los modelos.

**Resultados:**

De un total de 18,752 pacientes observados durante el periodo de estudio, se analizaron 6.727 (datos CRC) y 3.814 (datos BCCA). La mortalidad en los pacientes del grupo de datos CRC fue del 1,1% y en los del grupo de datos BCCA del 3,5%. Para el grupo de datos CRC, los modelos ACPGBI sobreestimaron significativamente la mortalidad a los 30 días, tanto en el modelo original como en el modelo revisado (O/E 0,17 y 0,21). La capacidad de los modelos para predecir correctamente el riesgo de mortalidad también fue limitada (test de Hosmer‐Lemeshow 23,1 y 22.9); sin embargo, el área bajo la curva ROC de ambos modelos fue de 0,88 (i.c. del 95% 0,85‐0,92) con una buena capacidad discriminatoria para clasificar a los pacientes que fallecían durante los primeros 30 días tras la cirugía. El modelo original ACPGBI recalibrado presentó un buen comportamiento para la predicción de riesgo (tasa O/E 1,06), pero no fue así en el caso del modelo revisado ACPGBI recalibrado (tasa O/E 0,99). En la validación externa con los datos BCCA, los modelos recalibrados subestimaron el riesgo de mortalidad a los 30 días (tasa O/E 3,06 y 2,98), mientras que los modelos ACPGBI original y revisado sobreestimaron el riesgo (tasa O/E 0,48 y 0,69, respectivamente). Todos los modelos mostraron una buena discriminación en las curvas ROC.

**Conclusión:**

Los modelos ACPGBI original y revisado sobreestimaron el riesgo de mortalidad a los 30 días. Se desarrolló un nuevo modelo, denominado modelo ACPGBI calibrado australiano o modelo ACACPGBI, cuya utilidad en la práctica clínica debe ser evaluada.

## Introduction

Colorectal cancer is the second most common cause of cancer‐related death after lung cancer in the developed world. About 16 400 new cases and 5500 deaths are registered in Australia every year^1^. Postoperative mortality from colorectal cancer varies across hospitals in Australia and New Zealand^2^.

In 2003, the Association of Coloproctology of Great Britain and Ireland (ACPGBI)^3^ derived a scoring system from a cohort of 4491 patients collected over 12 months (1999–2000) from 73 UK hospitals, for use specifically in surgical patients with colorectal cancer. The original ACPGBI score incorporates five operative variables: age, cancer resection, ASA fitness grade, Dukes' stage and urgency of surgery. A revised ACPGBI model was made available online in 2010^4^, and in 2011 was evaluated on 423 patients collected over 10 years (1997–2007) at a single public hospital in the UK as an accurate predictor of operative mortality after resection of colorectal cancer^5^.

The original ACPGBI model predicted the risk of postoperative death in a patient cohort from the UK; when the paper was originally published, this risk was 7·5 per cent^3^. However, the postoperative mortality rate in Australasia has been demonstrated in recent years to be less than 2 per cent^6^.

The aim of this study was to assess the original and revised ACPGBI models as predictors of 30‐day mortality in Australasian patients undergoing colorectal cancer surgery. A secondary aim was to determine whether adjustment or recalibration of these models based on data from Australasian participants could improve the prediction.

## Methods

Surgical data from patients with colorectal cancer were collected from six hospitals in Australia through three databases, and from hospital records. Assessment was performed based on intention to treat, and included all patients taken to the operating room for abdominal surgery for treatment of colorectal cancer. Data from patients undergoing surgery at the Cabrini and Alfred Hospitals in Melbourne, Victoria, between April 2006 and July 2015 were collected from hospital records and the Cabrini Monash colorectal neoplasia database^7^. Patient data were collected between January 2000 and December 2013 from Concord Hospital, Sydney, New South Wales, and between January 1996 and September 2014 from St John of God Subiaco, St John of God Murdoch and Freemantle hospitals in Perth, Western Australia. For most patients, the data were collected from practice and hospital individual‐patient records. If data relating to ASA grade were missing from patients treated at Cabrini and Alfred Hospitals, a single independent anaesthetist assigned a grade retrospectively after examining the patient's file. This data set from the six hospitals was termed the CRC data set.

An additional data set for further model testing, sourced from the Bi‐National Colorectal Cancer Audit (BCCA), comprised patients undergoing colorectal surgery between March 2007 and February 2016 from 79 hospitals across Australia and New Zealand. Patients treated in hospitals in the CRC data set were excluded from this additional data set to avoid duplication of patient data. The second cohort was termed the BCCA data set.

Patients with 30‐day mortality data were included in this study. Patients were subsequently included in risk model calculations when information for all required variables was available for each ACPGBI risk model. Variables required for the original ACPGBI model (2003) included: date of birth (age at surgery), ASA fitness grade, tumour stage (Dukes' stage A, B, C or D), date of surgery, urgency of operation (elective, urgent or emergency), whether the cancer was resected and 30‐day mortality (yes/no). The revised ACPGBI model (2010) also included operation/procedure type: right hemicolectomy; transverse colectomy; left hemicolectomy; sigmoid colectomy; subtotal/total colectomy; Hartmann's procedure; anterior resection; abdominoperineal excision of the rectum; examination under anaesthesia, laparotomy or laparoscopy only; or other. The majority of Australian and New Zealand hospitals use the Australian clinicopathological staging system (ACPS)^8^ and not the classical Dukes' system, which is strictly a pathological system of classification. In this study, Dukes' A, B, C and D were represented by ACPS stage I, II, III and IV respectively. Mortality was defined as death within 30 days of surgery, either in or out of hospital.

Ethical approval for this study was obtained from the Cabrini Human Research Ethics Committee (02‐10‐04‐06).

### Statistical analysis

To assess the prediction capability of the original and the revised ACPGBI models, first the 30‐day mortality risk was estimated for each patient in the CRC data set using the available parameter (score) for these models^3^, ^4^. Thereafter, observed mortality among the study patients was compared with estimated mortality according to the original and revised ACPGBI models. The calibration of these models was investigated using the Hosmer–Lemeshow goodness‐of‐fit test, which ascertains the ability of a model to predict the correct mortality risk^9^. For this test, the participants were divided into five groups according to predicted probability of death. Goodness‐of‐fit tests compared observed and estimated mortality for each group. The calibration was considered good if the estimated mortality did not differ significantly from the observed mortality (*P* > 0·050). The discriminatory power of the models was investigated by receiver operating characteristic (ROC) curve analysis, which showed how well the model classified patients who died within 30 days after surgery by comparing the predicted risk with the observed event. Area under the curve (AUC) values higher than 0·8 indicated good discrimination. A model could show good discrimination (separate those who died from survivors), but could sometimes overestimate or underestimate the risk (predicted probabilities disagree with observed proportions) with poor calibration.

The above models were also calibrated using the parameters available from logistic regression analyses using the same variables or risk factors used in the original and revised ACPGBI models. To do this, the CRC data set was first divided randomly into two groups, a developmental sample (CRC‐D) and a validation sample (CRC‐V), using the statistical software package. This was done irrespective of any set criteria, leading to an equal distribution of the data without bias. A recalibrated model (Australian calibrated model) was developed using CRC‐D and later validated internally (using tests for calibration and discrimination) on CRC‐V. Thereafter, the recalibrated models were validated externally using the BCCA data set. The calibration and discrimination of the original and revised ACPGBI models were also assessed in the BCCA data set. Data were analysed using Stata® version 14.2 (StataCorp, College Station, Texas, USA).

## Results

Of 18 752 patients observed during the study period (1996–2016), 10 541 were included in the present study. Specifically, data were collected from 2489 patients undergoing surgery at the Cabrini and Alfred Hospitals, 2211 from Concord Hospital, and 2125 from St John of God Subiaco, St John of God Murdoch and Freemantle Hospitals. Ninety‐eight patients (1·4 per cent of 6825) were excluded as data on a required data field were missing, leaving a total of 6727 patients for the CRC data set. The BCCA contained data on 11 927 patients collected from hospitals across Australia and New Zealand. Patients who did not have 30‐day mortality data available were excluded, and the remaining 3814 patients were included in the BCCA data set (*Fig*. *S1*, supporting information).

The median age of the 6727 patients in the CRC data set was 70 (range 19–100; mean(s.d.) 68·6(13·0)) years. Clinical characteristics are summarized in *Table* *1* and 30‐day mortality rates in relation to each variable are shown in *Table* *2*. Overall, 3·9 per cent of the patients had an ASA grade of IV or V, which was associated with the highest 30‐day mortality rate (8·8 per cent). The majority of patients (93·3 per cent) had elective surgery. Seventy‐five deaths were observed overall within 30 days after colorectal surgery, giving a mortality rate of 1·1 per cent. In univariable analysis, the strongest associations were seen between age over 95 years, ASA grade IV–V, Dukes' stage D, urgent surgery and 30‐day mortality.

**Table 1:** Clinical characteristics of patients in CRC data set

	No. of patients (*n* = 6727)*
**Age (years)** **†**	68·6(13·0)
< 65	2411 (35·8)
65–74	1861 (27·7)
75–84	1779 (26·4)
85–94	661 (9·8)
≥ 95	15 (0·2)
**ASA fitness grade**	
I	1227 (18·2)
II	3345 (49·7)
III	1883 (28·0)
IV–V	261 (3·9)
Missing	11 (0·2)
**Dukes' stage**	
A	1675 (24·9)
B	2173 (32·3)
C	1912 (28·4)
D	951 (14·1)
Missing	16 (0·2)
**Urgency of surgery**	
Elective	6274 (93·3)
Urgent	375 (5·6)
Emergency	78 (1·2)
**Operation type**	
Right hemicolectomy	2073 (30·8)
Transverse colectomy	62 (0·9)
Left hemicolectomy	184 (2·7)
Sigmoid colectomy	45 (0·7)
Subtotal/total colectomy	257 (3·8)
Hartmann's procedure	249 (3·7)
Anterior resection	3133 (46·6)
APER	411 (6·1)
EUA, laparotomy or laparoscopy only	112 (1·7)
Other	158 (2·3)
Missing	43 (0·6)

* With percentages in parentheses unless indicated otherwise;

† values are mean(s.d.). APER, abdominoperineal excision of the rectum; EUA, examination under anaesthesia.

**Table 2:** Thirty‐day mortality and univariable analysis of prognostic factors for mortality in CRC data set (6727 patients)

	30‐day mortality (%)	Odds ratio
**Age (years)**		
< 65	0·3	1·00 (reference)
65–74	0·5	1·62 (0·64, 4·12)
75–84	1·9	5·85 (2·70, 12·67)
85–94	3·3	10·34 (4·58, 23·34)
≥ 95	7	21·46 (2·51, 183·13)
**ASA fitness grade**		
I	0·1	1·00 (reference)
II	0·4	4·41 (0·57, 33·98)
III	2·1	25·93 (3·56, 188·97)
IV–V	8·8	118·48 (15·92, 881·51)
**Dukes' stage**		
A	0·3	1·00 (reference)
B	1·2	4·04 (1·55, 10·56)
C	0·8	2·64 (0·96, 7·28)
D	3·0	10·51 (4·05, 27·23)
**Urgency of surgery**		
Elective	0·7	1·00 (reference)
Urgent	7·7	11·87 (7·34, 19·20)
Emergency	3	3·73 (0·89, 15·65)
**Operation type**		
Right hemicolectomy	1·2	1·00 (reference)
Transverse colectomy	0	–
Left hemicolectomy	2·7	2·20 (0·83, 5·80)
Sigmoid colectomy	2	1·79 (0·24, 13·48)
Subtotal/total colectomy	1·2	0·93 (0·28, 3·09)
Hartmann's procedure	4·4	3·64 (1·78, 7·46)
Anterior resection	0·5	0·40 (0·22, 0·76)
APER	0·5	0·38 (0·09, 1·63)
EUA, laparotomy or laparoscopy only	1·8	1·43 (0·34, 6·11)
Other	4·4	3·65 (1·56, 8·55)

Values in parentheses are 95 per cent confidence intervals. APER, abdominoperineal excision of the rectum; EUA, examination under anaesthesia.

Among 3814 patients with 30‐day mortality data suitable for inclusion in the BCCA data set, the median age was 71 (range 22–107; mean(s.d.) 69·7(13·0)) years. Owing to missing information for some variables, 3236 patients were used in calculations regarding the original ACPGBI model and 3128 in those for the revised model. Thirty‐day mortality rates in relation to clinical characteristics in the BCCA data set are shown in *Table* *3*. Overall, 3·5 per cent of the patients had an ASA grade of IV or V, which was associated with the highest 30‐day mortality rate (24·8 per cent). The majority of patients (83·2 per cent) had elective surgery. There were a total of 133 deaths within 30 days after colorectal cancer surgery (mortality rate 3·5 per cent).

**Table 3:** Clinical characteristics and 30‐day mortality of patients in the Bi‐National Colorectal Cancer data set used for external validation of recalibrated original and revised ACPGBI models

	No. of patients (*n* = 3814)	30‐day mortality
**Age (years)**		
< 65	1253 (32·9)	10 (0·8)
65–74	1103 (28·9)	24 (2·2)
75–84	1069 (28·0)	51 (4·8)
85–94	369 (9·7)	46 (12·5)
≥ 95	20 (0·5)	2 (10)
**ASA fitness grade**		
I	565 (14·8)	1 (0·2)
II	1696 (44·5)	19 (1·1)
III	1169 (30·6)	71 (6·1)
IV–V	133 (3·5)	33 (24·8)
Missing	251 (6·6)	9 (3·6)
**Dukes' stage**		
A	847 (22·2)	15 (1·8)
B	1160 (30·4)	45 (3·9)
C	1081 (28·3)	42 (3·9)
D	354 (9·3)	23 (6·5)
Missing	372 (9·8)	8 (2·1)
**Urgency of surgery**		
Elective	3172 (83·2)	89 (2·8)
Urgent	323 (8·5)	17 (5·3)
Emergency	188 (4·9)	23 (12·2)
Missing	131 (3·4)	4 (3·1)
**Operation type**		
Right hemicolectomy	1321 (34·6)	64 (4·8)
Transverse colectomy	21 (0·6)	0 (0)
Left hemicolectomy	105 (2·8)	2 (1·9)
Sigmoid colectomy	26 (0·7)	4 (15)
Subtotal/total colectomy	185 (4·9)	8 (4·3)
Hartmann's procedure	138 (3·6)	10 (7·2)
Anterior resection	1407 (36·9)	21 (1·5)
APER	246 (6·4)	11 (4·5)
EUA, laparotomy or laparoscopy only	104 (2·7)	3 (2·9)
Other	84 (2·2)	6 (7)
Missing	177 (4·6)	4 (2·3)

Values in parentheses are percentages. ACPGBI, Association of Coloproctologists of Great Britain and Ireland; APER, abdominoperineal excision of the rectum; EUA, examination under anaesthesia.

### Performance of ACPGBI models

**Fig. 1:**
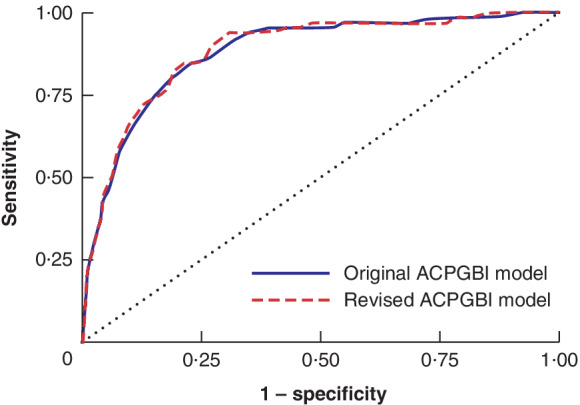
Discrimination of the original and revised Association of Coloproctologists of Great Britain and Ireland (ACPGBI) models on the CRC data set

*Table* *4* shows the observed and predicted 30‐day mortality for the original and revised ACPGBI models as well as the model performance (calibration and discrimination) in the CRC data set. Owing to missing information for some variables for predicting risk using the original and revised ACPGBI models, the analyses were restricted to 6700 and 6447 patients respectively. Both the original and revised ACPGBI models overestimated the risk of 30‐day mortality, but the rate estimated by the revised ACPGBI model was relatively closer to the observed rate. There was also a significant difference in the predicted 30‐day mortality rates derived using the original and revised ACPGBI models (*P* < 0·001) (*Table* *4*). In terms of calibration, the *P* value was significant (*P* < 0·050) in the Hosmer–Lemeshow test for both the original and revised models, suggesting lack of ability to predict 30‐day mortality risk among Australian patients. However, both ACPGBI models performed well in terms of discrimination, suggesting that they could classify patients who would die within 30 days after surgery. There was no difference in AUC for the two ACPGBI models *(Fig*. *1*).

**Table 4:** Performance of original and revised ACPGBI models on 30‐day mortality in CRC data set

					Calibration		Discrimination
	*n*	Observed mortality (%)	Estimated mortality (%)	O/E ratio	χ^2^	*P*	AUC
Original ACPGBI model	6700	1·1	6·7	0·17	23·1	< 0·001	0·88 (0·85, 0·92)
Revised ACPGBI model	6447	1·0	4·8	0·21	22·9	< 0·001	0·88 (0·85, 0·92)

Values in parentheses are 95 per cent confidence intervals. ACPGBI, Association of Coloproctologists of Great Britain and Ireland; O/E ratio, observed to expected mortality ratio; AUC, area under the curve.

**Fig. 2:**
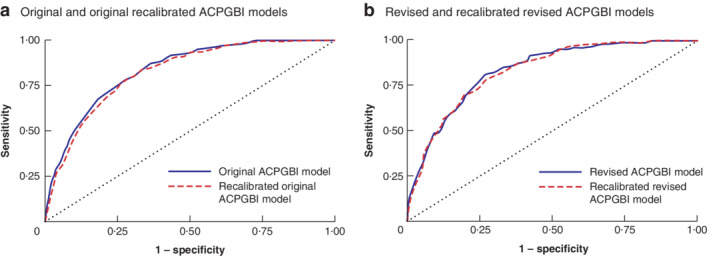
Discrimination of models on the Bi‐National Colorectal Cancer Audit data set **a** Recalibrated original Association of Coloproctologists of Great Britain and Ireland (ACPGBI) model on BCCA data set *versus* original ACPGBI model on BCCA data set (3236 patients). **b** Recalibrated revised ACPGBI model on BCCA data set *versus* revised ACPGBI model on BCCA data set (3128 patients).

### Performance of Australasian calibrated ACPGBI model

New parameters were developed from study patients (CRC‐D data set; 3349 patients for original ACPGBI model, 3222 for ACPGBI revised model) containing the same variables as the ACPGBI models to develop recalibrated original and revised ACPGBI models. The parameters obtained using the CRC‐D sample are summarized in *Table* *5*. Using the CRC‐D sample, both recalibrated models demonstrated good calibration and discrimination (*Table* *6*). When validated internally using the CRC‐V sample (3351 patients for original model, 3225 for revised model) only the recalibrated model based on the original ACPGBI model performed well for calibration. However, both recalibrated models demonstrated good risk prediction: O/E ratio 1·06, and 0·99 for original and revised models respectively (*Table* *6*). As the development and validation groups were sampled randomly from the respective data sets, there were no significant differences in mortality, urgency of surgery, ASA grade or age between the development and validation groups.

**Table 5:** Parameters obtained from CRC‐D for recalibration of original and revised ACPGBI models for risk prediction

	Score	
	Original ACPGBI model (*n* = 3349)	Revised ACPGBI model (*n* = 3222)
**Age (years)**		
< 65	0	0
65–74	0·75	0·58
75–84	1·49	1·35
85–94	1·99	1·80
≥ 95	3·03	3·19
**ASA fitness grade**		
I	0	0
II	13·37	14·74
III	14·12	15·51
IV–V	15·65	17·15
**Dukes' stage**		
A	0	0
B	0·17	0·23
C	0·16	0·13
D	0·47	0·32
**Urgency of surgery**		
Elective	0	0
Urgent	1·58	1·10
Emergency	–	
**Operation type**		
Right hemicolectomy		0
Transverse colectomy		–
Left hemicolectomy		–0·16
Sigmoid colectomy		1·32
Subtotal/total colectomy		–0·82
Hartmann's procedure		0·29
Anterior resection		–0·19
APER		–0·54
EUA, laparotomy or laparoscopy only		–0·11
		
Constant	–20·05	–21·22

ACPGBI, Association of Coloproctologists of Great Britain and Ireland. APER, abdominoperineal excision of the rectum; ASA, American Society of Anesthesiologists; EUA, examination under anaesthesia.

**Table 6:** Performance of recalibrated original and revised ACPGBI models on 30‐day mortality among Australian cohorts

					Calibration		Discrimination
	*n*	Observed mortality (%)	Estimated mortality (%)	O/E ratio	χ^2^	*P*	AUC
**For original ACPGBI model**							
Recalibrated model, CRC‐D data set	3349	1·1	1·1	0·98	3·1	0·378	0·90 (0·85, 0·95)
Recalibrated model, CRC‐V data set (internal validation)	3351	1·2	1·1	1·06	6·9	0·226	0·87 (0·82, 0·93)
Recalibrated model, BCCA data set (external validation)	3236	3·6	1·2	3·06	71·8	< 0·001	0·82 (0·79, 0·85)
Original ACPGBI model, BCCA data set	3236	3·6	7·6	0·48	25·0	< 0·001	0·83 (0·80, 0·86)
**For revised ACPGBI model**							
Recalibrated model, CRC‐D data set	3222	1·1	1·1	1·00	1·0	0·802	0·89 (0·84, 0·94)
Recalibrated model, CRC‐V data set (internal validation)	3225	1·0	1·0	0·99	48·5	< 0·001	0·87 (0·80, 0·93)
Recalibrated model, BCCA data set (external validation)	3128	3·6	1·2	2·98	63·7	< 0·001	0·83 (0·80, 0·87)
Revised ACPGBI model, BCCA data set	3128	3·6	5·2	0·69	26·8	< 0·001	0·83 (0·80, 0·87)

Values in parentheses are 95 per cent confidence intervals. ACPGBI, Association of Coloproctologists of Great Britain and Ireland; O/E ratio, observed to expected mortality ratio; AUC, area under the curve; CRC‐D and CRC‐V, development and validation data sets respectively derived from CRC data set; BCCA, Bi‐National Colorectal Cancer Audit.

From the BCCA data set, 3236 patients had complete data for calculations in the original ACPGBI risk model, whereas data from 3128 patients were available for the revised ACPGBI model. The recalibrated original and revised models underestimated the risk of 30‐day mortality in the BCCA data set (O/E ratio 3·06 and 2·98 respectively), but both original and revised ACPGBI models overestimated the risk of 30‐day mortality in the BCCA data set (O/E ratio 0·48 and 0·69 respectively) (*Table* *6*). However, ROC curve analysis showed that all the models showed similar good discrimination (AUC over 0·81) (*Table* *6* and *Fig*. *2*).

## Discussion

In this study, observed 30‐day mortality was compared with that predicted by the original and revised ACPGBI scoring systems in over 6000 patients undergoing resection for colorectal cancer in six hospitals across Victoria, New South Wales and Western Australia (CRC data set). Both ACPGBI models were found to overpredict mortality.

The observed 30‐day mortality rate was substantially lower than the rate predicted by the original ACPGBI model (1·1 *versus* 6·7 per cent). Similarly, observed 30‐day mortality was lower than that predicted by the revised ACPGBI model (1·0 *versus* 4·8 per cent). The ACPGBI models lacked good calibration, suggesting poor accuracy in predicting 30‐day mortality risk in an Australian cohort. However, discrimination was good for both models, indicating their ability to identify patients likely to die after surgery. Recalibration of the ACPGBI models to Australian patients resulted in both good calibration and good discrimination in the original model tested on validation samples of Australian patients (CRC‐V data set). Discrimination was also good for the revised model but not calibration, despite an O/E ratio of 0·99. This new model was termed the Australasian calibrated ACPGBI model, or ACACPGBI model.

Previous studies^10^, ^11^ showed that the original ACPGBI colorectal model tended to overestimate 30‐day mortality. One study^10^ suggested that the original ACPGBI model was largely inappropriate because of the few physiological variables included in the scoring system that might allow for patients receiving emergency surgery. The most commonly used systems are POSSUM^12^, Portsmouth POSSUM (P‐POSSUM)^13^ and colorectal POSSUM (CR‐POSSUM)^14^, and these were compared in a further study^11^. The original ACPGBI model gave more accurate predictions for elective procedures and surgery done by colorectal surgeons; however, of all the scoring systems analysed, prediction of overall mortality by CR‐POSSUM was closest to observed overall mortality^11^.

Further studies have assessed the different scoring systems in various countries, such as China, Turkey, the Netherlands and Denmark. A study^15^ from China compared multiple models, and concluded that CR‐POSSUM and the ACPGBI model were the most accurate predictors of postoperative mortality. Two Turkish studies^16^, ^17^ produced conflicting results, but showed the relative predictive value of both ACPGBI models, as well as the POSSUM, CR‐POSSUM and P‐POSSUM models. A study^18^ from the Netherlands concluded that the original ACPGBI model provided better prediction of mortality than P‐POSSUM, but CR‐POSSUM was the best for patients undergoing elective resections for colonic malignancy. A large study^19^ from Denmark comprising 21 370 patients tested the effectiveness of the original and revised ACPGBI models, and concluded that they were not suitable for predicting postoperative mortality in a Danish colorectal cancer population.

The mortality rates in the present study are lower than rates reported in the UK (6·5 and 5·7 per cent)^14^, ^20^, but are consistent with those from other international specialist centres^21^, ^22^, ^23^. Some of the patients from Australia and New Zealand in the BCCA data set are likely to have been treated by general surgeons, which may explain the higher observed mortality rates in this data set compared with the CRC one. The BCCA data set used in this study had a 30‐day mortality rate of 3·5 per cent, compared with 2 per cent for the BCCA as a whole across Australia and New Zealand^6^.

Potential problems with the methodological approach used in the development of scoring systems in general have been highlighted^24^. The objective of the ACPGBI project was to develop a mathematical model that predicts the probability of death after surgery for colorectal cancer^3^. There were two proposed uses of the model: in everyday practice and to compare the outcomes for colorectal cancer between multidisciplinary teams^3^. However, the original and revised ACPGBI models were developed over 17 and 10 years ago respectively, and may no longer be applicable to current surgical practice as different techniques and practices have been adopted or changed over time. For example, abdominoperineal resection techniques have progressed away from open surgery; an open operation was used for all such procedures in 2000 compared with less than 14 per cent currently^25^. Changes in surgical practice have also been evident in terms of the age at which colorectal cancer surgery is considered safe; surgery is now generally safe for those aged over 90 years^26^. In addition, many co‐morbidities directly influence outcomes, such as diabetes and high BMI^27^, ^28^.

According to the present results, all models, including the ACACPGBI model, showed very good discrimination, which is potentially helpful for understanding factors that influence the outcome following colorectal cancer surgery. However, the original and revised ACPGBI models overpredicted risk of death in this study, and so both models as they stand should be used with caution in Australia and New Zealand. The internally validated and revised ACACPGBI model will act as a robust tool for quality assurance and outcome comparison. Construction of a website to allow colorectal surgeons to enter data into the new ACACPGBI model is currently in progress.

## Supporting information


**Fig. S1** Study flow chart
