# Supplementary material for: Australasian ACPGBI risk prediction model for 30‐day mortality after colorectal cancer surgery
Source: BJS Open. 2020 Sep 28;4(6):1208–16. doi: 10.1002/bjs5.50356 (PMC7709373; doi:10.1002/bjs5.50356)

**BJS5_50356**

**Australasian ACPGBI** **risk prediction model for 30-day mortality after colorectal cancer surgery**

S. Wilkins, K. Oliva, E. Chowdhury, B. Ruggiero, A. Bennett, E. J. Andrews, O. Dent, P. Chapuis, C. Platell, C. M. Reid and P. J. McMurrick

**Fig. S1 Study flow chart**


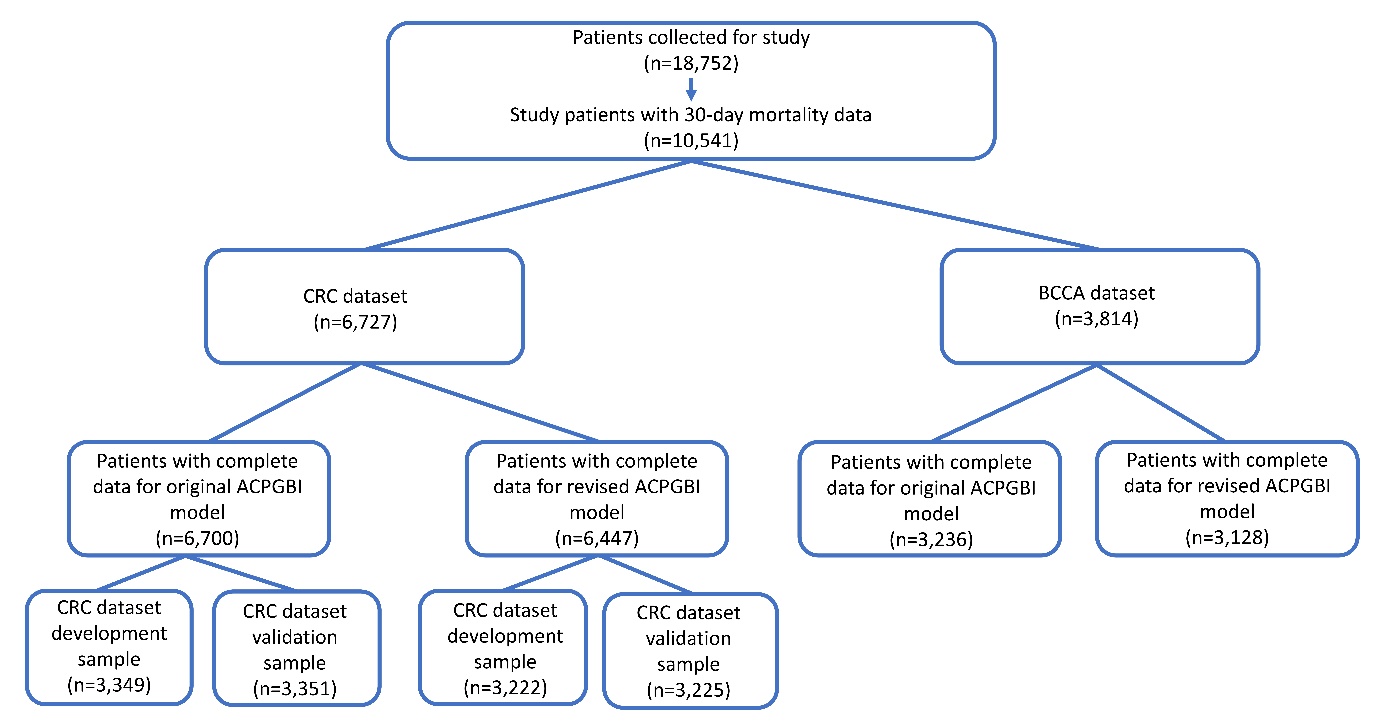

Supplement: Supplementary file 1 — Fig. S1 Study flow chart [file BJS5-4-1208-s001.docx]
